# Anti-Blast Performance of Polyurea-Coated Concrete Arch Structures

**DOI:** 10.3390/polym15051263

**Published:** 2023-03-02

**Authors:** Zhengyuan Yue, Jiannan Zhou, Xinli Kong, Ying Xu, Yishun Chen, Bo Wang, Yimiao Huang, Peng Wang

**Affiliations:** 1 State Key Laboratory for Disaster Prevention & Mitigation of Explosion & Impact, Army Engineering University of PLA, Nanjing 210007, China; 2 Department of Housing and Urban-Rural Development of Jiangsu Province, Nanjing 210036, China; 3 School of Civil and Transportation Engineering, Hebei University of Technology, Tianjin 300401, China

**Keywords:** concrete arch, polyurea strengthening, deformation, blast load, vibration damping

## Abstract

With the increasing number of violent terrorist attacks around the world, it is quite a common to improve the anti-blast performance of structures by reinforcing the exterior of the structure. In order to explore the dynamic performance of polyurea reinforced concrete arch structures, a three-dimensional finite element model was established by LS-DYNA software in this paper. Under the condition of ensuring the validity of the simulation model, the dynamic response of the arch structure under the blast load is investigated. Deflection and vibration of the structure under different reinforcement models are discussed. The optimum thickness of reinforcement (approximately 5 mm) and the strengthening method for the model were found by deformation analysis. The vibration analysis shows that the vibration damping effect of the sandwich arch structure is relatively excellent, but increasing the thickness and number of layers of the polyurea does not necessarily achieve a better vibration damping function for the structure. By reasonable design of the polyurea reinforcement layer and concrete arch structure, a protective structure with excellent performance of anti-blast and vibration damping can be created. Polyurea can be used as a new form of reinforcement in practical applications.

## 1. Introduction

Protective structures play an important role in reducing the number of casualties caused by explosion [1,2,3,4]. Traditional concrete structures have always been widely used in protection structures due to their high cost-effectiveness and nice mechanical properties. The dynamic response of concrete structures is more complicated when undergoing blast loading, and how to improve the anti-blast performance of concrete structures effectively has become a major concern for scholars worldwide [5,6,7,8]. Because of the low tensile strength of concrete, it is often undesirable to improve the anti-blast performance by increasing the volume of concrete alone [9,10], so reinforcing the outer surface of a concrete structure becomes an effective way of improving its anti-blast performance [11,12,13,14]. Studies in recent years have indicated that the application of fiber-reinforced polymer (FRP), which is more corrosion resistant, as reinforcement on protection structures is a reliable method [15,16,17,18,19].

The arch structure has been widely used in various underground protection structures. It can convert the vertical load into the horizontal thrust of the arch springing as the structure is in a compressed state, effectively reducing the tensile damage for the structure, making the arch structure more advantageous than the slab one in resisting the effect of blast load [6]. As a relatively common protective structure, the arch structure is widely used in cavern and tunnel protection structures, and it is necessary to investigate its protective performance under the effect of the blast load. Reinforced with FRP, the concrete arch structure is effective in controlling the cracking, reducing the deformation and improving the overall stability [20,21]. However, the FRP reinforced on the inner side of the arch tends to peel off when the force is very slight, which seriously affects the reinforcement effect of the FRP [22,23].

In order to further improve the blast resistance ability of concrete structures, it has been proposed to utilize coated polyurea [24,25], which has multiple properties such as anti-blast, anti-corrosion, abrasion and water resistance, is mainly applied in the form of coating on the concrete surface, due to its special chemical properties, and it forms a uniform coating of polyurea elastomer on the substrate within a short period of time. In this way, it is reinforced on the concrete structure and improves the anti-blast performance of the structure. As a new type of polymer material, polyurea also has advantages in environmental protection that other polymers cannot be matched [26,27]. For Polyurea coating has no organic solvents and no flammable gases to evaporate during the working process, it effectively protects the atmosphere and the health of the construction workers. After construction, the curing speed is fast, the performance is stable at a room temperature, and no harmful substances or odor are produced.

Compared with polyurea reinforced concrete slabs, FRP reinforcement has better static performance, while polyurea reinforcement has better performance under blast loading [25]. The protective effect of polyurea is revealed mainly on the non-explosive surface [28,29], where polyurea can participate in the stressing of the concrete structure effectively and the tensile stresses in the concrete are shared between the internal reinforcement and the external polyurea reinforcement layer [30,31]. The reinforcement of the polyurea leads to a great improvement in the anti-blast performance of the concrete slab and the structure becomes stronger with the increased thickness of the polyurea [26].

Liu et al. [29] confirmed that polyurea can effectively enhance the anti-blast performance of arch structures by conducting blast tests on semi-circular concrete arch structures with different reinforcement forms of polyurea, but no further analysis was put forward to investigate the protective mechanism of polyurea and the effect of coating thickness on the protective effect of arch structures. Blast tests are often complex and costly, and it is not advisable to carry out large-scale repetitive experiments, while the use of numerical simulation software to investigate the anti-blast performance of composite reinforced concrete structures is a valuable approach [32,33].

LS-DYNA is a software used by scholars to study the dynamic response of concrete structures commonly subjected to blast loads [34,35,36,37]. Carey N L et al. conducted a simulation study of a polyurea-reinforced concrete slab structure subjected to blast loading and selected different simulation models of damage for comparative analysis [33]. Several studies on the effect of mesh size on simulation results in different simulation models were carried out by Alãnon A et al. [38,39], and inductive conclusions were draw. Reifarth et al. further investigated the effect of mesh size selection on simulation results for reinforced concrete structures in finite element analysis under impact loading [40,41].

Previous scholars have mainly conducted simulation studies on polyurea-reinforced slab structures [28,30,33], but studies on polyurea-reinforced arch structures are fewer. In this study, in order to investigate the dynamic response of reinforced concrete arch structures after polyurea strengthening, anti-blast experiments were carried out on the prepared arch structures. Focusing on reinforced concrete arch structures and polyurea, the results of strengthening of polyurea on macroscopic damage to the structures were investigated. Using the LS-DYNA software R11.0., the deformation and vibration of the arch structure under different models of reinforcement are investigated, and a more comprehensive analysis of the arch structure under blast load is conducted. These works provide an economical and reasonable design basis for improving the anti-blast performance of arch structures in protective engineering.

## 2. Specimen Preparation and Materials

After the concrete structures undergoing the blast load, the dynamic response time of the structure is usually short in duration, and it is impractical to observe the experimental phenomena at close range during the explosion. In order to obtain a better understanding of the damage of the arch structure and to predict the dynamic response of the arch structure, analysis using finite elements is a common method. In this study, the finite element model is built using HYPERWORK software 2021 and calculated with LS-DYNA.

### 2.1. Geometrical Model Description

**Figure 1:**
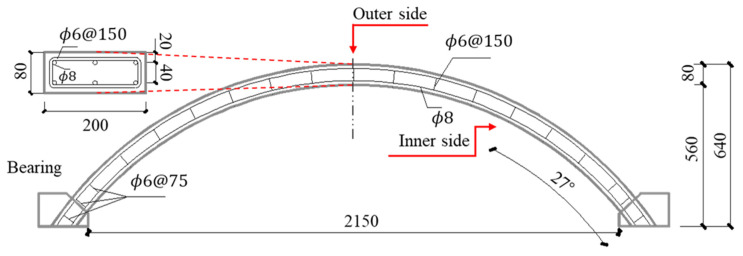
Reinforced concrete arch structure (mm).

The center angle of the arch was 108°, the outer diameter of the arch was 1445 mm, the inner diameter was 1365 mm, the thickness of the arches was 80 mm, the width was 200 mm, and the vector span ratio was 0.26. In the concrete structure, the used longitudinal reinforcements had a diameter of 8 mm, with stirrup bars of 6 mm in diameter. Three longitudinal forced bars were arranged on the outer side and three longitudinal stress bars were arranged on the inner side. The stirrup bars at arch springing were encrypted, which were arranged at 75 mm intervals, and the rest of the stirrup bars were arranged at 150 mm intervals. The stirrup bars were located on the outer side of the longitudinal steel bar and had a constrained effect on the longitudinal steel bar. The overall reinforcing bar layout is shown in Figure 1. The polyurea was coated at the inner side of the concrete arch structure. Table 1 lists the physical properties of concrete, steel and polyurea. The mass ratio of cement: medium sand: gravel: water was 30:35:50:15. In the process of pouring concrete, a batch of standard cylinder specimens were made. The relevant physical parameters of concrete were measured after 28 days of curing.

**Figure 2:**
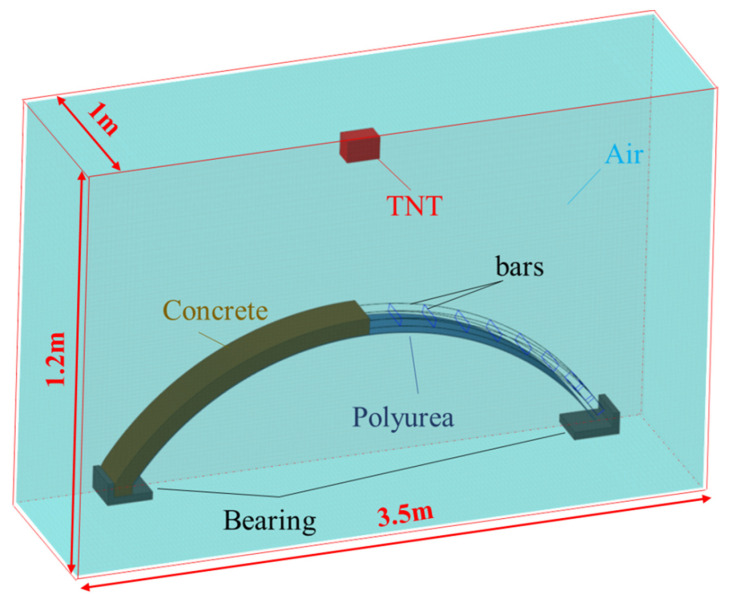
Finite element model.

In the process of analyzing performing finite element, the size of the mesh has a large impact on the result of the calculation: the finer the mesh is divided, the more accurate the outcome of the calculation is [38]. According to previous publications on mesh convergence studies [38,39], it can be concluded that in simulations of structures subjected to blast loads with similar working conditions, a parallel hexahedral mesh of 18 mm for concrete and 50 mm beam elements for bars are the best sizes to ensure the convergence of the structure calculated, and are in accordance with those used by other authors [40,41]. However, in order to achieve a more accurate observation of macroscopic damage, the mesh size was suitably reduced in this simulation. A parallel hexahedral mesh with 10 mm sides was used for the concrete arch structure, and the bars elements were arranged above the nodes of the concrete elements. Each concrete model consisted of 44,160 solid elements and 2296 beam elements. The finite element model is shown in Figure 2.

### 2.2. Geometrical Model Description

The numerical simulation required the modelling of a total of six materials including bars, concrete arch, polyurea, TNT and air, of which the bars mainly consisted of stirrup bars and longitudinal bars. Modelled by BEAM elements, the bars are first understood as one-dimensional line segments with no cross-sectional area in which the different line segments are joined together and are subsequently given section properties with the SECTION_BEAM keyword. Concrete arch structures are modelled by 3D Lagrange solid elements. When dealing with the relation between bars and concrete, the possible cementation and sliding displacements between the bars and concrete are often ignored and the bars are considered to be embedded and reinforced in the solid elements of the concrete [42,43]. The polyurea is modelled by 3D Lagrange solid elements in this paper.

In practical experiments, polyurea may show slight debonding after blast loading. In order to be to simulate the actual working conditions as accurately as possible, the contact between polyurea and concrete is defined by using the CONTACT_SURFACE_TO_SURFACE keyword. Air and TNT are simulated using the ALE mesh, with the control card CONSTRAINED_LAGRANGE_IN_SOLID, to enable the inflow of air and the effect of the shock wave from the explosion on the structure. In this keyword, NQUAD, CTYPE, DIREC and MCOUP are some of the more important parameters. The value of NQUAD depends on the density of the fluid and solid mesh. The size of the ALE mesh in this study is a square hexahedron with a side length of 20 mm, where the value of NQUAD is chosen to be 3. For the occurrence of erosion of Lagrangian solid element, CTYPE = 5 is more appropriate. Using DIREC = 2 makes the calculation more stable. In case of MCOUP = 0, the TNT and the air can be combined to impact on the structure. The advantage of the ALE element is that it can exist independently of the 3D solid element, avoiding mesh distortion during the subsequent solving process.

In this numerical simulation study, instead of building a monolithic bearing model, the bearing was only built at the foot of the arch and constrained by the BOUNDARY_SPC card so that to control the dimensions of freedom of the bearing to ensure compliance with the experimental working conditions. The contact between the concrete arch and the bearing is defined by the CONTACT_SURFACE_TO_SURFACE card, which controls the vertical displacement at the base of the arch effectively. Up to this point, the simulation conditions are essentially identical to the bearing constraints in the blast tests.

In addition, due to the limitation of modelling area, the BOUNDARY_NO_REFLECTING card was used to set the non-reflective boundary on the outer surface of the model to simulate the test conditions in the infinite air area. In practical conditions, after the blast load, the shock wave is applied to the structure by air transfer, which often consumes a very short period of time and the gravitational acceleration in the model has almost no effect on the structure. In order to achieve a more reasonable model, the effect of the structure’s gravity on the results is not considered in this simulation.

### 2.3. Material Model

#### 2.3.1. Bars

The bars used in this experiment are hot rolled ribbed steel bars. The steel is a typical linear elastic material, the stress–strain is linear at the beginning of loading and fracture occurs when the stress increases to the tensile strength. The MAT_PLASTIC_KINEMATIC material card was used to demonstrate the constitutive model of the bars [38,39], which is a non-linear plastic material model which is made under the consideration for the effect of strain rate effects. The physical properties of the steel bars are represented in Table 1.

#### 2.3.2. Concrete

Dynamic response of concrete structures under blast loading is a generally complex process. The strain rate effect is a key consideration when choosing the constitutive model for concrete. The material model of concrete chosen for this paper was MAT_CSCM_CONCRETE (continuous surface cap model) from LS-DYNA software, which was more frequently used for numerical simulations of the mechanical properties of concrete materials under blast load and impact load [37,44,45] and had the significant advantage of simple input parameters. In addition, the model has its own strain rate effect and element eroding. Setting the IRATE parameter to 1 turns on the strain rate effect of the material and setting the ERODE parameter to 1 defines that the eroding element would be deleted. In numerical simulations, the damage to concrete can be represented macroscopically by the erosion damage which comes with the material model. In spite of the shortcomings of failure analysis by element erosion, this method is indeed commonly used [46]. The material parameters for the concrete in this model are shown in Table 2. The value of UNIT is 4 when it is in international system units.

#### 2.3.3. Polyurea

**Figure 3:**
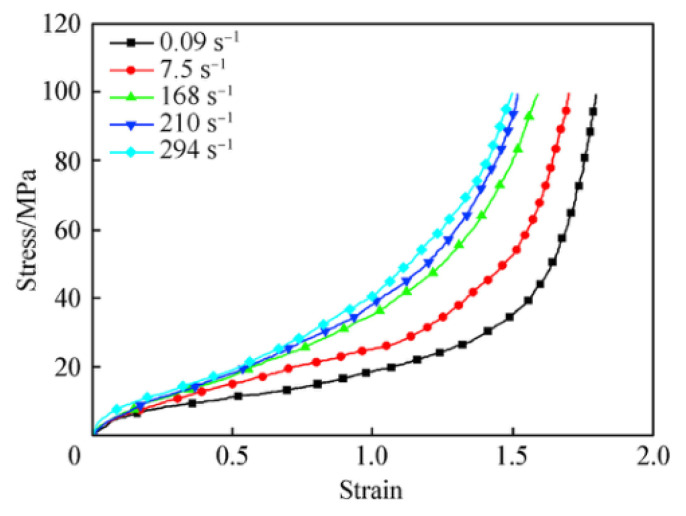
Stress−strain curve for polyurea at various strain rates. [26].

Polyurea has a complex microstructure, which is influenced by strain rate greatly, resulting in a more complex dynamic response under blast loading. Traditional stress–strain curves are difficult to represent the dynamic response. In order to obtain the appropriate properties of polyurea more accurately, the MAT_PIECEWISE_LINER_PLASTICITY material card was chosen for this research, combined with the curve of polyurea stress–strain influenced by strain rate, to work together to simulate the force on the polyurea [26,27,28], as shown in Figure 3. The properties of the polyurea in this study are listed in Table 1.

#### 2.3.4. TNT

The production of TNT blasting can be simulated with the MAT_HIGH_EXPLOSIVE_BURN material card in conjunction with the EOS_JWL equation of state. The relevant parameters involved in this material model include density of TNT *ρ*, velocity of wave D, and blast pressure P. The equation of state involves parameters including the coefficients *A, B, R_1_, R_2_, ω,* and the density of initial internal energy *E_0_* and the relative volume V. The parameters are taken as shown in Table 3, and the pressure P generated by the explosion can be expressed as

(1) $$P=A\left(1-\frac{\omega }{R_{1}V}\right)e^{-R_{1}V}+B\left(1-\frac{\omega }{R_{2}V}\right)e^{-R_{2}V}+\frac{\omega E_{0}}{V}$$

#### 2.3.5. Air

Air can be simulated by the MAT_NULL material model with the equation of state of EOS_LINEAR_POLYNOMIAL. The simplified expression for the pressure of air is

(2) $$\;P=C_{1}+\left(C_{4}+C_{5}\mu \right)E$$

where *C*_1_, *C*_4_, and *C*_5_ are the correlation coefficients. *μ* = *ρ* / *ρ*_0_ − 1, which can be calculated from the current density of air ρ and the initial density of air *ρ*_0_, where *E* is the density of the internal energy of the air. The values of the parameters involved in air are shown in Table 4.

## 3. Explosion Test and Simulation Validation

### 3.1. Experiment Test

**Figure 4:**
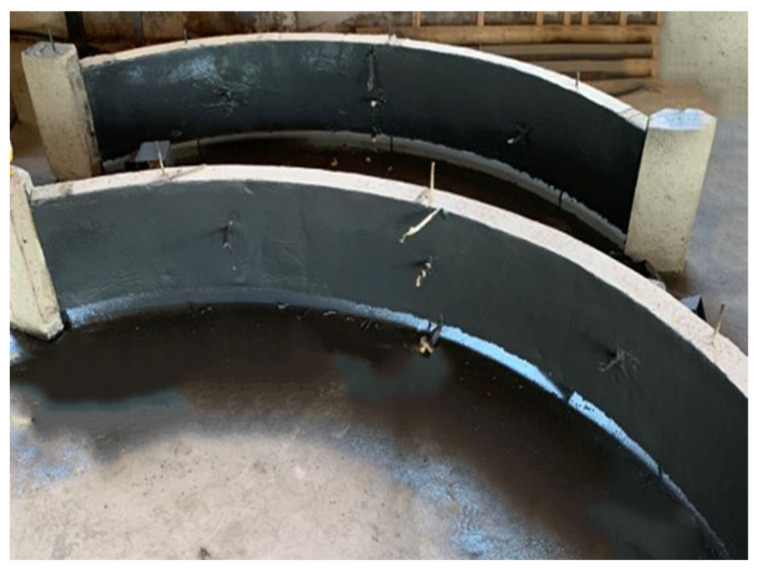
Polyurea-reinforced arch structures.

**Figure 5:**
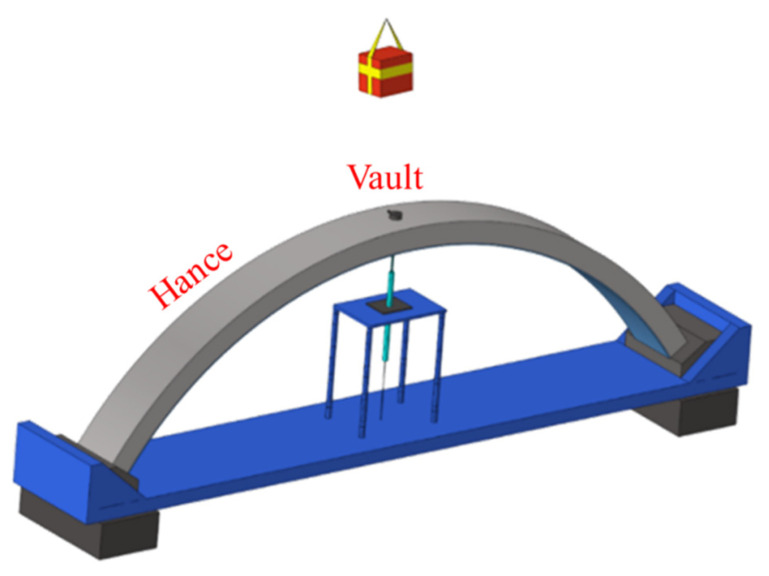
Arrangement of anti-blast experiment.

This sub-section verifies the validity of the numerical model through experimental results. The composite arch structure after polyurea coated is shown in Figure 4. A model diagram of the experimental arrangement of the arch structure for anti-blast is shown in Figure 5. Table 5 shows the test scheme for this anti-blast experiment.

### 3.2. Simulation Validation

#### 3.2.1. Damage

**Figure 6:**
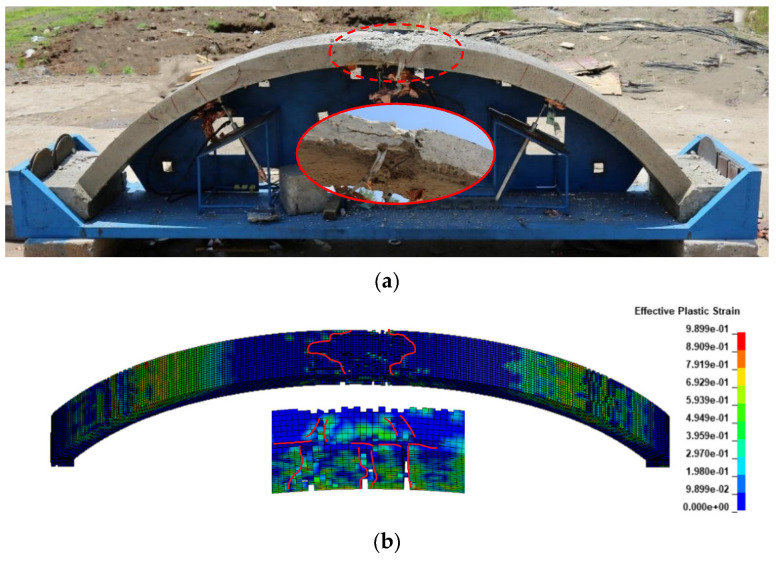
Damage to A2 (**a**) Experimental (**b**) Simulation.

**Figure 7:**
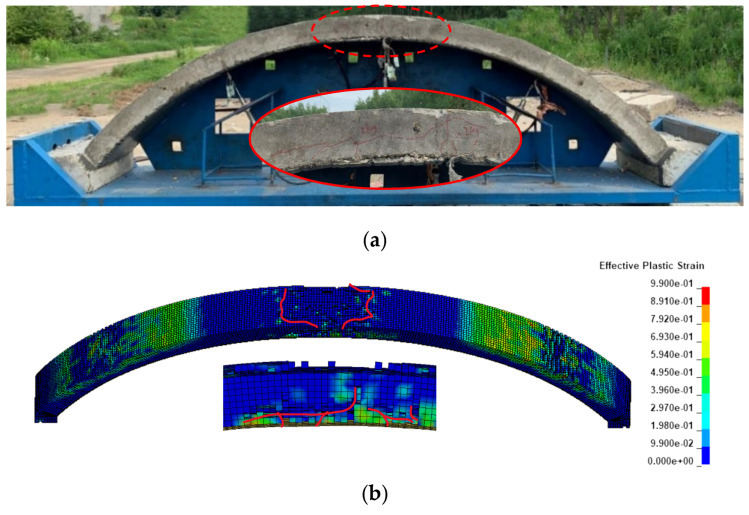
Damage to the A3 in (**a**) Experiment, accessed on 13 July 2022 (**b**) Simulation.

Component A2 (Figure 6) and A3 (Figure 7) were selected for analysis of damage under blast load of 2 kg TNT with stand-off distance of 0.85 m. Figure 6a shows the damage to component A2 after the explosion in the blast test. The figure shows that the concrete on the outer side of the vault was crushed and that at the inner side of the vault appeared to be spalled, with some of the bars exposed together with penetration cracks. The results of the simulation are shown in Figure 6b, where the concrete elements on the outer side of the vault are deleted due to reaching the failure strength, and the concrete on the inner side of the vault shows two tension cracks and exposing bars. The comparison in this figure shows that the simulated results are basically consistent with the macroscopic damage state obtained from the experimental results at this working condition.

The damage of A3 strengthened by polyurea is shown in Figure 7a. The concrete on the outer side of the vault shows only minor damage, with a slight portion of the concrete crushed and a diagonal shear crack. Meanwhile, the polyurea on the inner side of the vault shows slight debonding and the vault maintains a good integrity, as is shown in Figure 7b. It is suggested that the concrete on the outer side of the vault appears slightly crushed, and significant diagonal shear cracks appear at the bottom of the arch. A few cracks appear at the hance, and the overall arch structure still has good integrity. The results of the simulation show a high agreement with the crack development observed in the experiments.

This model can be helpful for effective analysis of the damage of concrete structures and polyurea strengthened concrete structures after blast load. The comparative test shows a high reliability of this model for describing the damage of polyurea strengthened concrete arch structures under blast load.

#### 3.2.2. Reflective Pressure

**Figure 8:**
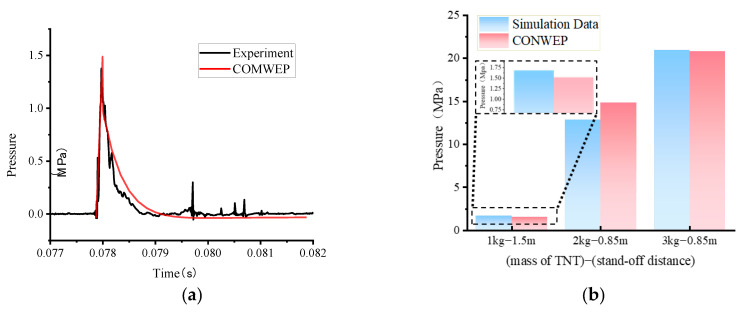
Reflected pressure in experiment, simulation, CONWEP. (**a**) Reflected pressure of the vault under blast load of 1 kg TNT with stand-off distance of 1.5 m. (**b**) Peak of pressure at the vault.

In order to ensure that the data measured by the experiments and simulations are accurate and reliable, the anti-blast experiment was conducted by selecting member A1 for the trial test explosion and measuring the reflected pressure on the surface of the vault, which was compared with the empirical CONWEP formula and the reflected pressure on the vault measured by the simulations. In blast testing, the comparison of the resulting pressure data with the empirical CONWEP formula is a common method to ascertain experimental accuracy [29,47]. In the experiment, the measured reflected pressure at the outer side of the vault under 1 kg TNT with stand-off distance of 1.5 m was compared with the numerical simulations and CONWEP results calculated as shown in Figure 8. As shown in Figure 8a,b, the peak reflected pressure measured by the experiment is 1.38 MPa and the peak reflected pressure calculated by CONWEP is 1.52 MPa, both of which are in good agreement with the load time and within a reasonable tolerance. After the explosion, the pressure at the vault reaches its maximum instantaneously, indicating that the sensor has been affected by the explosion pressure at this time until the shock wave dissipates and then approaches a value of zero again. Possibly caused by the shock wave reflected from the ground, two abrupt peaks can be pointed in the pressure time curve measured by the test.

Due to the large mass of TNT used in this experiment, the reflected pressure sensor could not capture the data well, and only one set of data from the reflected pressure sensor was measured. As a result of the above analysis, it was found that the pressure data measured by the experiment met the desired conditions of the explosion test and that the data and phenomena derived from the experiment were reliable.

In order to ensure the reliability of the numerical simulation results, the peak pressure of the concrete element at the top of the arch was selected after the calculation was completed and compared with the peak pressure of the CONWEP empirical formula. Figure 8b shows a comparison of the peak of reflected pressure at the top of the arch from the simulation and the CONWEP empirical formula. Numerical simulation results indicate that the peak pressure in the vault withstanding blast load of 2 kg TNT with stand-off distance of 0.85 m is 12.87 MPa and the peak pressure calculated by the CONWEP empirical formula is 14.82 MPa. The peak pressure in the vault under blast load of 3 kg TNT with stand-off distance of 0.85 m is 22.98 MPa. Tolerances for peak pressures are all kept within acceptable ranges.

The data of the reflected pressure at the vault show that the numerical simulation, the experiment and the CONWEP empirical equation are in good agreement, thus indicating that the blast test meets the expected requirements and that the simulated model and the experimental data measured have a strong reliability.

#### 3.2.3. Acceleration

**Figure 9:**
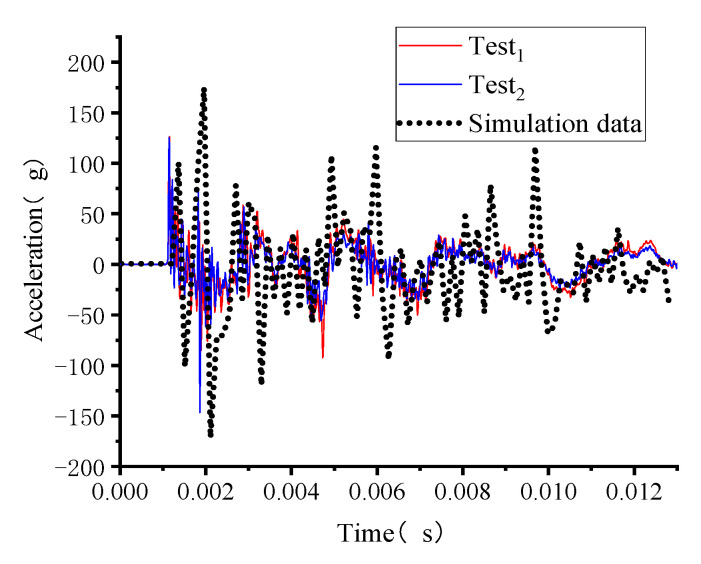
Dynamic acceleration measured at the inner side of the vault of member B1.

Acceleration is often an important parameter for judging the vibration of a structure [30,48]. Dynamic accelerations on the inner side of the vault were measured in this experiment. To ensure the accuracy of the experimental data, two different types of acceleration sensors (Test_1_, Test_2_) were arranged at the same measurement point on the inside of the vault. The dynamic acceleration data obtained from experimental and numerical simulations withstanding blast load of 0.1 kg TNT with stand-off distance of 1 m are shown in Figure 9, indicating that the two different dynamic acceleration curves from the same measurement point are highly consistent, which confirms the validity of the data from this experiment. In addition, the simulated results have similar peaks and fluctuation frequencies to those measured in the experiment. Probably due to the difference between the cast particle sizes of the concrete elements used in the experiments and the simulations, there is a slight difference between the result of experimental measurements and the result of simulations. However, the simulation results are within the acceptable tolerance range. It is demonstrated that this simulation model can be used to measure the dynamic acceleration of a structure under the present conditions.

## 4. Further Numerical Investigation 

### 4.1. Deformation Analysis

**Figure 10:**
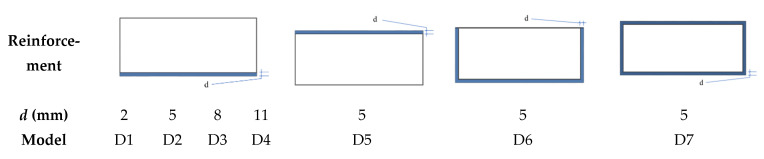
Anti-blast simulation scheme. (*d* is the thickness of polyurea).

In order to find the most cost-effective way to improve the anti-blast performance of the concrete structure, the strength of the concrete (40 MPa, 60 MPa), the thickness of the polyurea and the location of the polyurea reinforcement were varied in the numerical simulation study in this paper (Figure 10) as a way to further analyze the anti-deformation ability of the structure to test its reliability against blast load.

**Figure 11:**
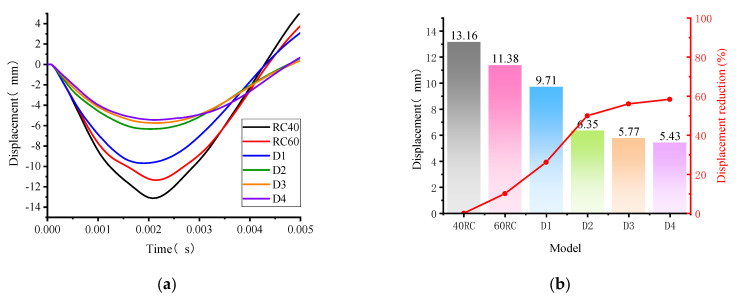
Displacement (**a**) Displacement curve of the vault (**b**) Peak displacement and percentage reduction.

In this study, the dynamic response of a concrete arch structure withstanding blast load of 0.2 kg TNT with stand-off distance of 0.2 m was calculated by simulation. As is shown in Figure 10a, the displacement-time curves for the top of the arch obtained by varying the concrete strength (40 MPa, 60 MPa) and the reinforcement thickness (models D1, D2, D3, D4). where the direction of displacement downwards is the negative. After the blast load, the vault experienced a downward displacement first and a bounce back took place after reaching the peak displacement. Changing the concrete strength and the thickness of the polyurea reinforcement is an effective way to reduce the deformation degree of the arch structure, but it does not subversively change the response time of the structure to reach peak displacement. Figure 11b suggests that the concrete strength increases from 40 MPa to 60 MPa and the arch displacement of the arch structure decreases by 10.12%.

In order to investigate the effect of the reinforcement thickness of polyurea on the anti-blast performance of the structure, the dynamic response of the composite arch structure (concrete strength of 40 MPa) was calculated for the same working conditions. Figure 10b indicates that the displacement of the vault decreases by 26.21% (D1), 50.07% (D2), 56.15% (D3) and 58.51% (D4) with increasing the thickness of the polyurea. However, when the thickness of the polyurea is increased from 5 mm to 8 mm or 11 mm, the anti-blast performance of the structure does not increase in a large percentage, which could be influenced by the strength of the concrete itself and the way in which the polyurea reinforcement is applied. Therefore, when the concrete strength is 40 MPa and the requirements of protection are not too strict, the reinforcement of 5 mm polyurea at the bottom of the arch structure can effectively improve the blast resistance of the structure, which is an economical method in practical engineering.

The polyurea is reinforced on the concrete in the form of coating. In addition to reinforcement at the inner side of the arch, this paper also investigates the effect of other forms of reinforcement on the anti-burst performance of the structure, such as models D5, D6 and D7.

**Figure 12:**
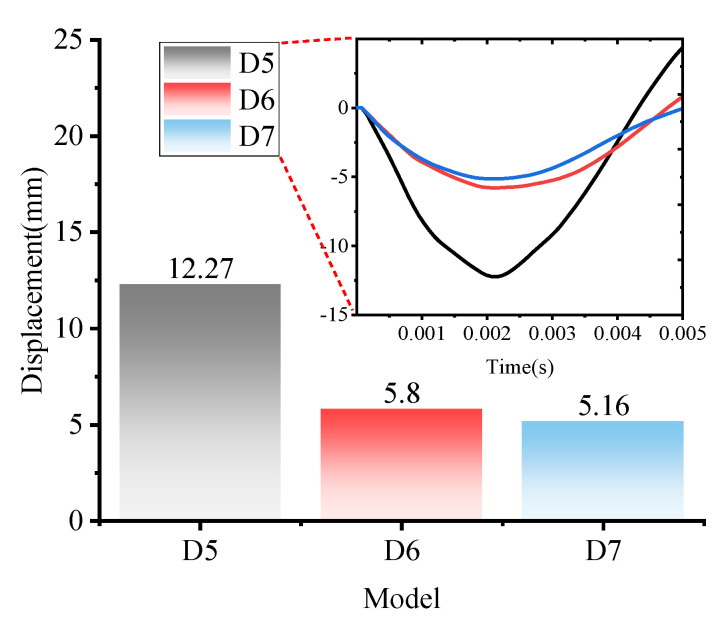
Displacement peaks of model D5, D6 and D7 and curves.

As is shown in Figure 12, when the polyurea reinforcement is on the outer side of the vault, the maximum vertical displacement of the vault measured is −12.27 mm, which is 6.67% less than the displacement of the RC structure, and the maximum vertical displacement of the vault measured by models D6 and D7 are −5.80 mm and −5.16 mm, compared to the displacement of the RC structure, is reduced by 55.93% and 60.79%, respectively.

A preliminary analysis of the deformation of the arch structure under the different working conditions illustrated above shows that increasing the strength of the concrete can restrain the deformation of the arch under blast loading. However, reinforcement with polyurea is a practical method if the deformation of the structure is to be restrained substantially. In the case of polyurea reinforcement with a defined thickness, the best form of reinforcement to improve the anti-blast performance is the enclosed-coating reinforcement (model D7), and the second is the semi-coating (model D6). Model D5 is the most inefficient form for the arch structure is mainly under compression on the outer side of the arch, and the main protection mechanism of polyurea is to restrain the generation of tension cracks. The reinforcement, existing in the form of a sacrificial layer, is not effective enough on the outer side of the vault so cannot inhibit the deformation of the structure considerably.

### 4.2. Vibration Analysis

#### 4.2.1. Acceleration (Y-Direction, i.e., Vertical)

In the first portion of this study, the effect of polyurea reinforcement on the anti-blast performance of arches was investigated, showing that polyurea is advantageous to improve the anti-blast performance of the structures. Nonetheless, more efforts are needed to investigate the effect of polyurea reinforcement on the vibration damping effect of the structure. The amplitude of change in acceleration is a factor that describes the vibration of the structure, which is the sum of the absolute value of the maximum acceleration and the absolute value of the minimum acceleration. The smaller the value, the better the anti-vibration effect of the structure.

**Figure 13:**
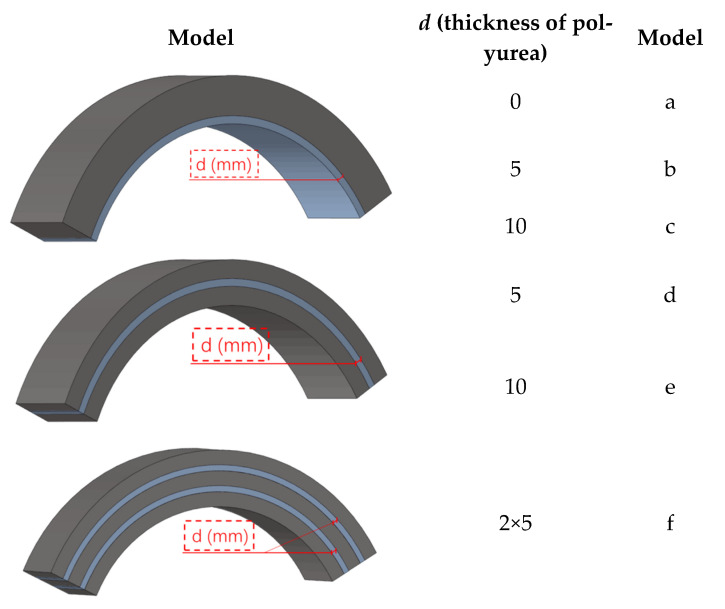
Vibration damping plan for arch structure. (*d* is the thickness of polyurea).

**Figure 14:**
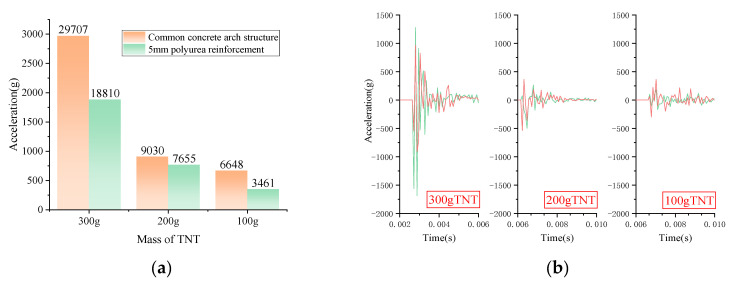
Acceleration in the Y-direction, i.e., vertical (**a**) Maximum variation of acceleration at the inner side of the vault. (**b**) Dynamic acceleration curve of the vault. (Three different conditions).

**Figure 15:**
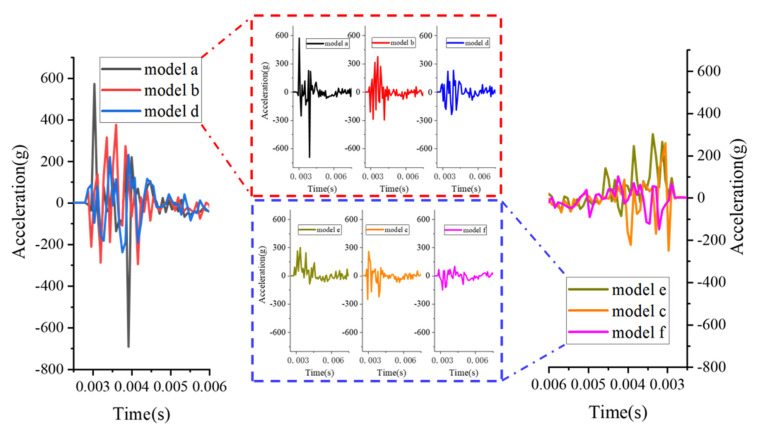
Acceleration (Y-direction, i.e., vertical) in the hance with understanding blast load of 0.3 kg TNT with stand-of distance of 1 m.

In order to further study the dynamic response of the structure under more demanding conditions, the dynamic acceleration of the arch under different models (Figure 13) was simulated under the premise of ensuring the accuracy of the above simulation model (Figure 9), and the dynamic acceleration was obtained as shown in Figure 14, Figure 15 and Figure 15. The models described in the subsequent articles are all shown in Figure 13. Figure 14 shows the maximum variation of acceleration resulting from the structure being subjected to blast loads of different mass of TNT in Figure 14a and the dynamic curve of acceleration in Figure 14b. From Figure 14a,b, it can be seen that as the mass of TNT increases, the measured variation in acceleration gradually tends to increase, and the variation in acceleration within the vault of the polyurea-reinforced arch members is significantly less than that of the unreinforced.

If the polyurea is placed in the mid-arch, one arch is divided into several arches, reinforced with concrete in the form of a sandwich. As in Figure 13 models d, e and f, which result in a composite sandwich structure. The form of the composite material combination affects the performance of the structure to a considerable extent, with the structural form changing and the dynamic response changing accordingly [49,50].

With the increase in mass of TNT, the dynamic response at the vault is more sensitive and the noise fluctuation cannot respond well to the dynamic response of the structure. Therefore, the acceleration at the vault will not be selected for analysis. The vertical acceleration in the hance is smaller than in the vault, but it is also effective in describing the difference in dynamic response between the different structures.

As shown in Figure 15, the vertical acceleration fluctuations on the inner side of hance are obtained by subjecting the blast load of 0.3 kg TNT with stand-off distance of 1 m. The graph clearly indicates that the thickness of the polyurea and the position of the polyurea in the arch structure have a large influence on the peaks and fluctuations of the acceleration. From the comparison analysis between model b and model c, it can be seen that the maximum variation of acceleration at the hance is diminished due to the reinforcement of the polyurea, which is consistent with the results of the previous analysis in Figure 14. In addition, as the thickness of polyurea increases, the structure has better effect of vibration damping.

Besides the thickness of the polyurea reinforcement, changing the position of the polyurea can also be used as a way of influencing the vibration of the structure. Figure 15 shows that the peak acceleration of the structure is suppressed effectively when the polyurea is reinforced in the mid-arch structure, which means the vibration damping effect of models d, e and f is better than that of model b and c, indicating that when the polyurea reinforcement is placed in the middle of the concrete arch structure, there is a better vibration damping effect on the structure and it is better than polyurea reinforcement at inner side of the arch.

In order to further investigate the influence of polyurea in the middle of the arch on the vibration damping effect of the structure, the thickness of the polyurea and the reinforcement method are also considered in this paper. The thickness of the polyurea was not only increased to 10 mm (model e), but the reinforcement pattern was also changed.

By dividing the 10 mm polyurea into two 5 mm parts, each reinforced in the middle of the arch structure (model f) to evaluate the impact on vibration damping of structural. As is indicated in Figure 15, with the thickness of the polyurea sandwich increasing to 10 mm (model e), the acceleration on the inner side of the hance is mostly positive and the hance is accelerating upwards at this stage. Increasing the thickness of the polyurea and reducing the thickness of the concrete making the composite structure less stiff and more likely to be displaced in one direction rather than vibrating around the value of zero. The maximum variation of model d is not significantly different compared to model e. The absolute value of the peak acceleration for both models shows that the absolute value of the peak acceleration for model d is approximately 2000 m/s^2^, which is less than the 3000 m/s^2^ for model e, and the vibration damping effect of model d is better than model e for similar amplitude of variation. With flexible polyurea, the structure becomes more susceptible to a large deformation as the thickness of polyurea increases, making it more likely to vibrate in one direction. As for maximum amplitude of acceleration and peak of acceleration, model f shows the best effect of vibration damping. The two layers of polyurea dampens the vibration of the arch structure effectively, and the maximum variation of acceleration and peak of acceleration obtained are significantly less than the other models.

#### 4.2.2. Resultant Acceleration

**Figure 16:**
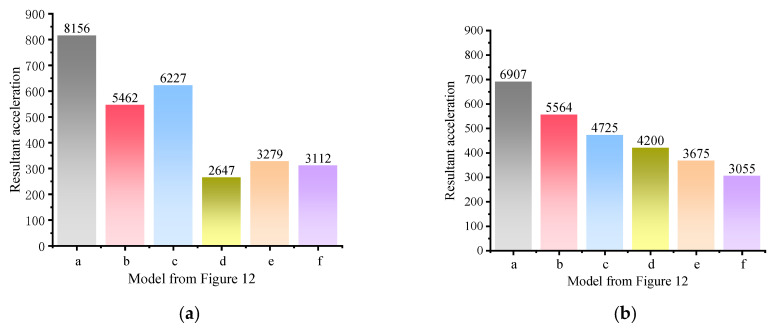
Resultant acceleration at the hance (**a**) inner side of the hance (**b**) outer side of the hance.

Figure 16 shows the resultant acceleration on the inner side of the hance. This parameter considers not only the acceleration in one direction, but the scalar sum of the acceleration in all three directions. Apparently, in Figure 16a, from the perspective of the resultant acceleration, the sandwich structure has a better effect of vibration damping than the polyurea reinforcement at the inner side of the arch. Changing the thickness of the interlayer and the form of interlayer reinforcement has less effect on the resultant acceleration of the structure and may even has a negative influence for the increased thickness of the interlayer causes the structure to vibrate in a horizontal direction, resulting in a slight increase in the resultant acceleration, but the sandwich structure is still the most effective form of vibration damping in the paper.

The acceleration on the inner side of the hance is analyzed mainly to study the dynamic response at the bottom of the arch, while the arch structure is often cracked on the outer side of the hance, so the information presented by the dynamic acceleration on the outer side of the hance is also very crucial. Figure 16b shows the dynamic peak of resultant acceleration on the outer side of the hance for different reinforcement model. The graph shows that the resultant acceleration on the outer side of the hance is not significantly different from the resultant acceleration on the inner side of the hance for the first three reinforcement models (model a,b,c). However, in the last three conditions (model d, e, f), i.e., the sandwich structure, the resultant acceleration on the outer side of the hance appears somewhat different from the inner side. The resultant acceleration on the outer side of the hance of the sandwich structure is slightly higher than the inside. It may be that the upper arch absorbs the vast majority of the energy when the sandwich structure is subjected to blast loading, and that the combined effect of the upper arch and the polyurea weakens the impact of the shock wave effectively on the down arch, so that the peak acceleration of the upper arch is slightly higher than that of the down arch.

In this subsection, the acceleration of the structure is analyzed to explain the effect of different reinforcement models on the vibration damping effect of the structure. The sandwich structure has better energy absorption ability and properties of vibration damping than the external bonding, and polyurea absorbs the energy generated by the blast load effectively, which diminishes the influence of shock waves on the lower arch. One arch structure is divided into three arches and separated by two layers of polyurea, with the middle arch structure being reinforced above and below with polyurea. For such structures subjected to blast and impact load, the effect of vibration damping is stronger than in the model of Figure 13 (model d, e). Although such sandwich structures are more effective in vibration damping, the anti-blast performance of which cannot perform as well as models b and c (Figure 13) due to the weaker tensile properties of the concrete and the reduced thickness of individual arches, which render the structure to be more prone to cracking [51,52].

## 5. Conclusions and Future Work

In this study, the dynamic responses and damages of polyurea-coated concrete arch structures under blast load are revealed through anti-blast experiments and numerical simulations. The software LS-DYNA was used to analyze the deformation of the composite arch structure under different strengthening models, and the dynamic accelerations obtained from the simulations were further analyzed to reveal the effect of polyurea on the vibration damping of the structure. It is concluded that:

1. In this study, the failure pattern of concrete arch structures after blast load was dentified by experiment. The arch structure reinforced by polyurea is mainly fractured in oblique shear at the vault, indicating that the polyurea strengthening has shifted the damage pattern from bending damage to shear damage.
2. By comparing the results of the numerical simulations with the data attained in current experiments, it is obvious that the simulation model is capable of predicting the damage of the arch structure and the dynamic data, providing a reference for future simulations of the dynamic response of concrete arch structures under blast loading.
3. Polyurea in strengthened condition can lower the degree of deformation of the vault effectively, and increasing the thickness of the polyurea can further suppress the deformation. However, when the polyurea is strengthened to an extreme level, it becomes too thick (over 5 mm), and the anti-blast performance of the structure will not be enhanced significantly. When the thickness of the polyurea is defined, the enclosed-coating form of reinforcement has the best anti-blast properties. When polyurea is strengthened on the outer side of the arch, the arch structure has the worst explosion resistance.
4. The thickness and location of the polyurea reinforcement have a considerable influence on the effect of vibration damping of the structure. Polyurea reinforcement in the middle of arch is a relatively effective method of vibration damping; however, increasing the thickness of the polyurea cannot guarantee a better vibration-damping effect.

Polyurea-coating on the inner side of arch structure is of great help to improve the anti-blast performance of reinforced concrete arch. More research on the bonding performance and debonding mechanism of polyurea concrete under explosive loading is needed in order to extend the dynamic response of composite strengthened structures. Apart from the dynamic response of the structure, the constitutive model of polyurea can also be one of the directions for future research in order to improve the understanding of the reinforcement mechanism of polyurea.

In addition, the constitutive model of CSCM used in this paper can appropriately simulate common concrete structures, but it does not have a high reliability for the UHPC (ultra-high performance concrete). The lack of studies in this orientation curbs the utilization of new materials in engineering applications. Therefore, it is necessary to calibrate the constitutive model of CSCM [45] to expand its applicability to a wide range of materials. The calibrated parameters of CSCM appear to expand the application of UHPC in the field of impact engineering.

## Figures and Tables

**Table 1:** Physical properties of concrete, steel and polyurea.

Concrete					Reinforcing Bar				
Unconfined compressive strength		Tensile strength		Elastic modulus	Ultimate tensile strength	Yield strength	Elastic modulus	C	P
24.6 MPa		1.95 MPa		21 GPa	455 MPa	335 MPa	200 GPa	40.4 s^−1^	5
**Polyurea**									
Density (g/cm)	Shore hardness A (°)	Poisson rate	Fracture strain	Tensile strength (MPa)	Tear strength (*N*/mm)	Abrasive resistance (750 g, 500 r)	Gelation time (s)		
1.2	90	0.4	350%	18	55	30 mg	10–20		

**Table 2:** Parameters of the concrete model.

*ρ* (kg/m^3^)	IRATE	ERODE	*f _c_* (MPa)	UNIT
2400	1	1.0	24.6	4

**Table 3:** Parameters of TNT and equation of state parameters.

*ρ* (kg/m^3^)	*D* (m/s)	*P_cj_* (Gpa)	*A* (Gpa)	*B* (Gpa)	*R*_1_	*R*_2_	*ω*	*E*_0_ (J/m^3^)	*V*
1631	6718	18.5	374	3.75	4.15	0.9	0.35	7 × 10^9^	1.0

**Table 4:** Parameters of air and equation of state parameters.

*ρ* (kg/m^3^)	*C*_1_	*C*_4_	*C*_5_	*E*_0_ (J/m^3^)	*V*
1.293	−1 × 10^5^	0.4	0.4	2.5 × 10^5^	1.0

**Table 5:** Anti-blast test scheme.

Arch	Thickness of Polyurea	TNT (kg)	Stand-off Distance (m)	Scaled Distance (m/kg^1/3^)
A1	5 mm	1	1.50	1.50
A2	0	2	0.85	0.67
A3	5 mm	2	0.85	0.67
B1	5 mm	0.1	1.00	2.15

## Data Availability

The data presented in this study are available on request from the corresponding author.
